# Culture of endometrial epithelial cells collected by a cytological brush in vivo

**DOI:** 10.3168/jdsc.2021-0189

**Published:** 2022-03-16

**Authors:** Cecilia Constantino Rocha, Felipe Alves Correa Silva, Thiago Martins, Marcela G. Marrero, John J. Bromfield, John Driver, Thomas Hansen, Lilian Oliveira, Mario Binelli

**Affiliations:** 1 Department of Animal Sciences, University of Florida, Gainesville 32611; 2 Department of Biomedical Sciences, College of Veterinary Medicine and Biomedical Sciences, Colorado State University, Fort Collins 80521; 3 Department of Pathology, College of Veterinary Medicine, University of Georgia, Athens 30602

## Abstract

In cattle, mechanistic studies of endometrial function rely on cell lines or primary culture of cells harvested postmortem. Understanding the endometrial physiology in dairy cows is essential, because approximately 50% of pregnancies are lost in the first 3 wk of gestation for unknown reasons. The objective was to validate an in vivo, minimally invasive, and estrous cycle stage–specific method to obtain endometrial luminal epithelial cells for culture. The uterine body of 26 cows was sampled using a cytology brush (cytobrush) 4 d after estrus. The viability of cells was measured by flow cytometry (80% live cells) and epithelial identity was determined by anti-vimentin and anti-cytokeratin immunofluorescence and quantitative PCR for *KRT18* and *VIM*. A pool of cells from 15 animals was passaged 4 times in culture until confluent and then treated with 0, 0.1, 1, or 10 ng/mL of recombinant bovine interferon-tau (rbIFN-τ). The relative expression of transcripts related to IFN-τ signaling (*IFNAR1*), early (*IRF2*) and late (*ISG15*, *OAS1*) response to IFN-τ stimulus, and other IFN-τ–stimulated genes (*CCL8*, *CXCL10*, and *FABP3*) was measured by quantitative PCR. The relative expression of *KRT18* transcripts was similar across passages; the relative expression of *VIM* increased at passage 2, and *IFNAR1* transcripts decreased in cultured compared with that in fresh cells. The relative expression of *ISG15*, *OAS1*, *CCL8*, and *FABP3* increased in response to rbIFN-τ. In conclusion, culture of endometrial luminal cells collected by cytobrush was feasible, generating a monolayer enriched in epithelial cells, and therefore constitutes a novel model by which to study endometrial luminal epithelial cell function, including responses to IFN-τ.

## Highlights

• Endometrial cytology (cytobrush) yields samples enriched in luminal epithelial cells.
• Cells collected via cytobrush proliferate in culture.
• Recombinant bovine IFN-τ stimulates the expression of IFN-stimulated genes in cultured cells collected via cytobrush.

## Graphical Abstract

**Figure:**
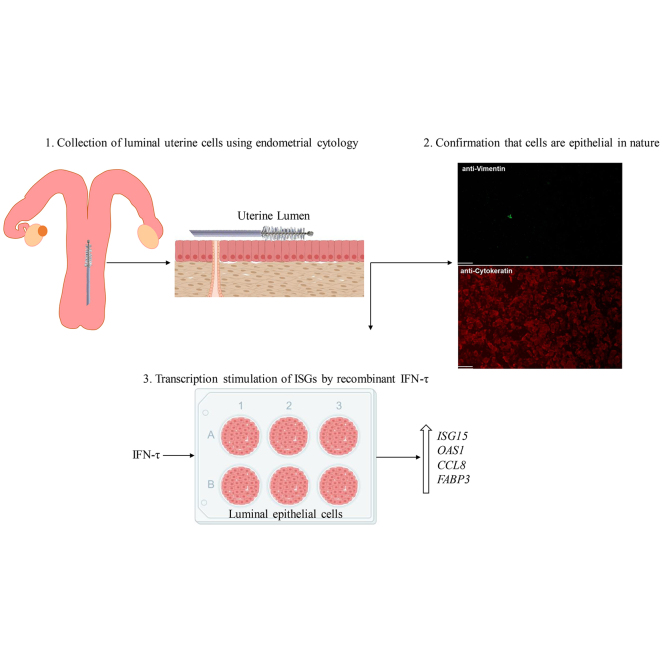
**Summary:** Understanding endometrial physiology in dairy cows is essential, as approximately 50% of pregnancies are lost in the first 3 weeks of gestation for unknown reasons. This work validated an approach to culture and propagate luminal endometrial cells harvest by a minimally invasive tool, endometrial cytology (cytobrush). (1) Cells were collected at a specific stage of the estrous cycle; (2) fresh cells were evaluated regarding their identity by immunofluorescence; and (3) functionality of the system was demonstrated by inducing transcription of IFN-stimulated genes (ISGs) in response to treatment with recombinant IFN-τ.

Reproductive success is limited by embryonic mortality and implantation failure (Diskin and Sreenan, 1980). In dairy cows, around 50% of pregnancies are lost in the first 3 wk of gestation (Wiltbank et al., 2016). Successful pregnancy establishment depends on autocrine, paracrine, and endocrine events that occur at the maternal–fetal interface (Oliveira et al., 2008; reviewed by Rocha et al., 2021). Before implantation, the main maternal component interacting with the embryo is the luminal endometrium. Inadequate endometrial receptivity is one of the key barriers to a successful gestation (Gray et al., 2001). Therefore, uterine health and a functional endometrium are crucial factors in the dairy industry. The endometrium is composed of a single layer of columnar epithelial cell lines, the uterine lumen, and is separated from the stromal cells by a basal membrane (Ohtani et al., 1993). The luminal epithelial cells are the most proximal target for embryo-derived pregnancy factors, such as IFN-τ (Asselin et al., 1997).

Bovine uterine epithelial cells (**BUEC**) are used widely as a model to study the physiology of pregnancy in dairy and beef cows (Binelli et al., 2001; Forde et al., 2011; Talukder et al., 2017; Sakai et al., 2018). Cells are mostly from immortalized commercial cell lines or obtained postmortem from the slaughterhouse (Forde et al., 2011; Rizo et al., 2019; Sponchiado et al., 2020). However, there are limitations associated with these methods: they require slaughtering of animals to harvest tissues and cells; most of the time, tissues originate from a slaughterhouse and animals are from unknown breeds and health status. Stage of the estrous cycle or pregnancy can only be estimated and, for primary cultures, isolation of the different cell types requires multiple enzymatic digestions that may influence cell function (Taghizadeh et al., 2018). Here, we describe a method that uses a minimally invasive methodology to harvest BUECs in vivo at a specific stage of the estrous cycle in cattle and to culture them without enzymatic digestion. To accomplish this, we used a cytological brush, which is minimally aggressive to the uterus, can be used on successive collections over the estrous cycle without changing estrous cyclicity (Batista et al., 2019), and does not interfere with fertility (Martins et al., 2022). Our model for the current study used beef cows with a view to extrapolating the results to dairy cows as the uterine histology of both is identical. To test the validity of this model, we measured the relative expression of transcripts associated with IFN-τ signaling after treating BUECs with recombinant IFN-τ (**rbIFN-τ**).

Animal welfare guidelines and handling procedures were approved by the Institutional Animal Care and Use Committee from the University of Florida under protocol number IACUC-202009814. The estrous cycles of multiparous *Bos taurus indicus*–influenced cows (n = 38) were synchronized using an injection of prostaglandin F_2α_ analog [dinoprost tromethamine, Lutalyze; Zoetis (25 mg)]. Cows observed in estrus (n = 26) were included in the study. Four days after estrus, luminal epithelial cells were collected in vivo with a cytology brush (Cytobrush; Disposable cytology sampling brush; Viamed Ltd.) as described by Cardoso et al. (2017). Day 4 was selected because of the intense cell proliferation at this cycle stage (Mesquita et al., 2015), which is essential for propagation in culture. Briefly, the cytological brush was coupled to the tip of a conventional AI gun covered by an AI sheath and a sanitary sheath. The whole apparatus was introduced in the female reproductive tract. When the cervix was reached, the sanitary sheath was disrupted. After clearing the cervix, the brush was exposed, and the operator rotated the apparatus 5 times; then, the brush was retracted in the AI sheath to remove it from the reproductive tract. After collection, brushes containing cells were stored in Dulbecco's modified Eagle medium/F12 (cat. no. 11330057, Gibco/ThermoFisher Scientific), supplemented with 10% fetal bovine serum (cat. no. 26140079, Gibco/ThermoFisher Scientific), 3% penicillin-streptomycin (cat. no. 091670049, MP Biomedicals/ThermoFisher Scientific), and 2% amphotericin B (Fungizone; cat. no. ICN1672348, MP Biomedicals/ThermoFisher Scientific). Cells were kept at room temperature for immunofluorescence (n = 8) and cell culture (n = 15) and on ice for flow cytometry (n = 3).

Cell viability was determined by flow cytometry. Brushes were rinsed with PBS (Ca^2+^ and Mg^2+^ free) to remove adhered cells. Cells were pelleted at 700 × *g* at 4°C for 5 min and washed with PBS. After adjusting the concentration to 10^6^ cells/mL, cells were treated with rat IgG (2 μg/mL, Sigma-Aldrich) for 10 min to reduce nonspecific binding and subsequently incubated with LIVE/DEAD Fixable Near-IR Dead Cells Stain Kit (L34976; Invitrogen/ThermoFisher Scientific), following the manufacturer's instructions. Unstained cells from each animal were used as controls. Cells were assayed in an Attune NxT Flow Cytometer (Invitrogen/ThermoFisher Scientific). The proportion of dead cells (positive staining) was identified after gating on viable single cells. The results were analyzed using FlowJo software (Treestar).

Cell type was identified by immunofluorescence. Fresh cells were isolated approximately 1 h after collection, as described for flow cytometry. The cell pellet was dispersed and air-dried on glass slides, and then fixed and permeabilized with ice-cold acetone for 10 min. Slides were washed in 1% normal goat serum (S-26; Sigma-Aldrich) diluted in PBS and blocked for 30 min in 10% goat serum. Next, the slides were stained for dual-color immunofluorescence analysis using rabbit anti-vimentin (cat# D21H3, 2 μg/mL; tagged with Alexa Fluor 488 conjugated, ThermoFisher Scientific) and mouse anti-pan cytokeratin (cat# MA110325 10 μg/mL; tagged with Alexa Fluor 594 conjugated, Thermo/Fisher Scientific) diluted in antibody staining buffer. After incubation, slides were washed, and nuclear labeling was performed using ProLong containing 4′,6-diamidino-2-phenylindole (**DAPI**; cat. no. P36962; Invitrogen). The slides were examined using a Leica DM2500 LED microscope with filters 02 (DAPI), 03 [fluorescein isothiocyanate (FITC)], and 15 (rhodamine) at 20× magnification. Digital images were acquired using Leica LASX software and a high-resolution Leica DMC6200 digital camera. Five random fields were captured for cell counting (TCS SP5, Leica). The proportion of cells (detected by DAPI) that were positive for each cell marker was determined using 100 cells per animal. The remaining nonpermeabilized cells were used to prepare additional slides using Diff-Quik coloration (Pascottini et al., 2015).

Culture and propagation of cells were performed as described previously (Sponchiado et al., 2020). Briefly, fresh cells from 15 animals (10^5^) were plated in multiple wells of 6-well plates using 1 plate per animal (hereafter, plated samples). After reaching confluence, cells were split for passaging (passage 1). Cells from 2 to 3 animals were combined in a pool. Four pools were generated and propagated to confluence in P175 flasks (passage 2). Cells were subsequently harvested, cryopreserved, and thawed as described previously (Sponchiado et al., 2020), and then plated in P75 flasks and grown to confluence (thawed/passage 3). Next, cells were again dispersed and plated in 6-well plates (passage 4). Throughout the propagation process, cells from each step were saved for future quantitative (**q**)PCR analysis (fresh, plated, passage 1, passage 2, thawed, and passage 4). At each passage, cDNA was synthesized from total RNA extracted from ~10^6^ cells. Real-time-PCR was used to measure the relative abundance of transcripts for *IFNAR1* (Sponchiado et al., 2020), *KRT18*, and *VIM* (Cardoso et al., 2017) using iTaq Universal SYBR Green supermix (cat. no. 1725124, Bio-Rad/ThermoFisher Scientific) in a Bio-Rad CFX Connect light cycler (BioRad) as described previously (Rocha et al., 2020). The geometric average of the reference genes *PPIA* and *ACTB* was used to calculate relative expression, using the Pfaffl method (Pfaffl, 2001).

To evaluate the responsiveness of BUECs to rbIFN-τ, confluent passage 4 cells (10^5^/well in 12-well plates) were incubated with serum-free medium for 4 h (starvation). Starved cells were treated with 0, 0.1, 1, or 10 ng/mL of rbIFN-τ (provided by Dr. Thomas Hansen, Colorado State University) in serum-free medium for 24 h and harvested for qPCR analysis. The abundance of transcripts related to IFN-τ signaling (*IFNAR1*), early (*IRF2*; Shirozu et al., 2017) and late (*ISG15*, *OAS1*) response to IFN-τ stimulus (Sponchiado et al., 2020), and other IFN-τ–stimulated genes (*CCL8*, *CXCL10*, and *FABP3*; Sakumoto et al., 2017; Schabmeyer et al., 2021) was measured by qPCR. The experiment was repeated 3 times, and each treatment was tested in triplicate.

The effect of passage or the effect of concentration of rbIFN-τ on relative expression of each gene was evaluated by one-way ANOVA using PROC MIXED in SAS software (version 9.4; SAS Institute Inc.). When the effect of passage was significant, means were separated using Dunnett's test, considering fresh cells as the control group. When the effect of concentration of rbIFN-τ was significant, means were separated by the Tukey test. The results are presented as means ± SEM. We considered significance at *P* ≤ 0.05 and approaching significance when 0.05 < *P* ≤ 0.1.

Flow cytometry analysis of fresh cytobrush samples demonstrated that approximately 80% of cells were viable (Figure 1a). We found that 91 ± 4.9% and 5 ± 3.3% of cells, respectively, were positive for cytokeratin and vimentin by immunohistochemistry (Figure 2). Cytokeratin and vimentin colocalized in 4 ± 1.8% of cells, and 0.5 ± 0.2% of cells expressed neither marker.

**Figure 1:**
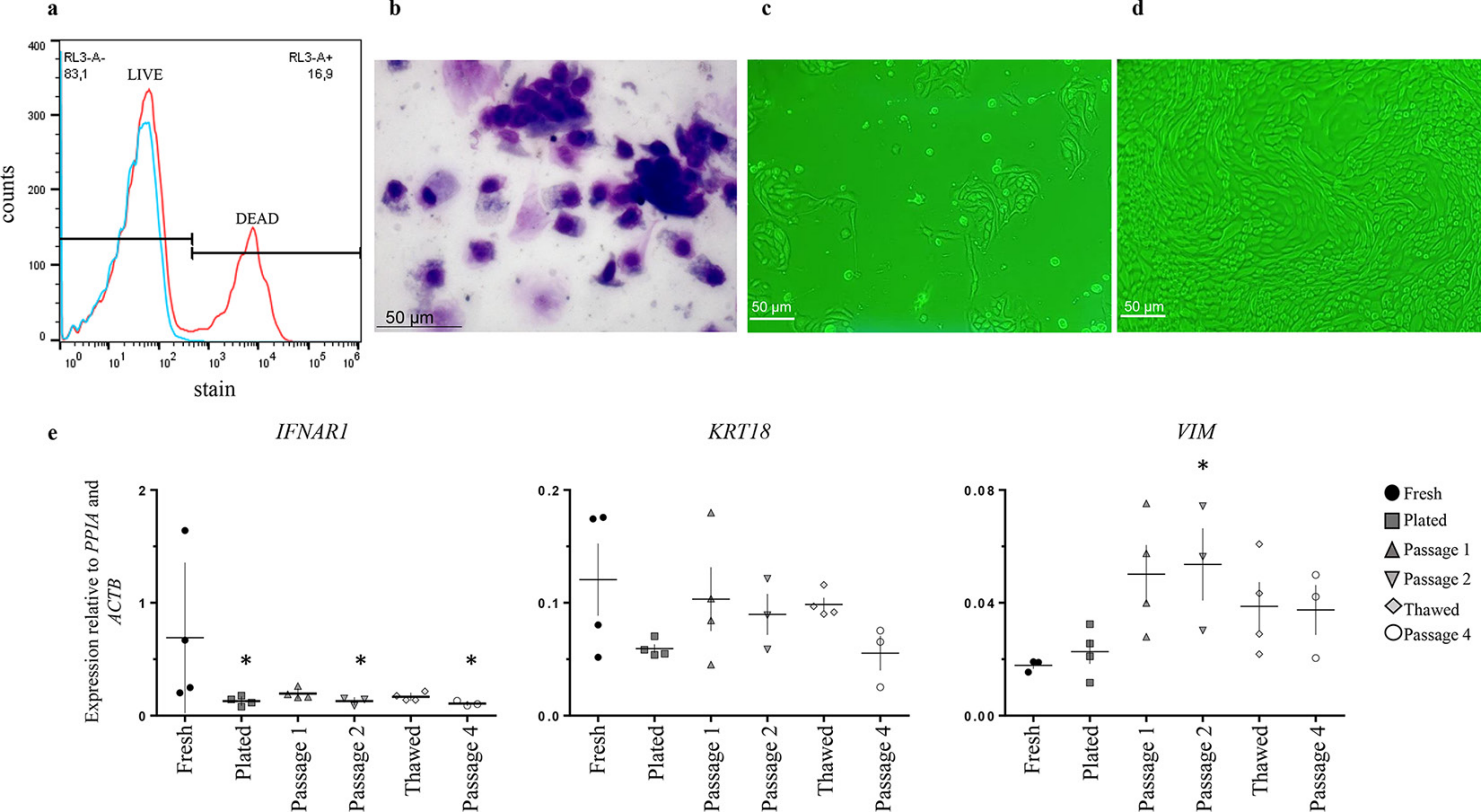
Proportion of live cells, cellular morphology, and select relative expression of cytobrush-derived endometrial luminal cells. (a) Representative histogram of the proportion of live cells measured by flow cytometry. The y-axis shows the number of cells, and the x-axis indicates the amount of color detected (blue = unstained sample; red = stained sample); (b) optical microscopy of cells harvested immediately after collection by cytobrush (Diff Quick staining); (c, d) optical microscopy of cultured cytobrush-derived cells in early (Fresh) and late (Passage 4) stages of confluence; (e) relative abundance of *IFNAR1*, *KRT18*, and *VIM* during culture progression. Each data point represents one pool (means ± SEM; n = 4 pools, each composed of cells from 2 to 3 animals). *Significantly different from control (Fresh; *P* < 0.05).

**Figure 2:**
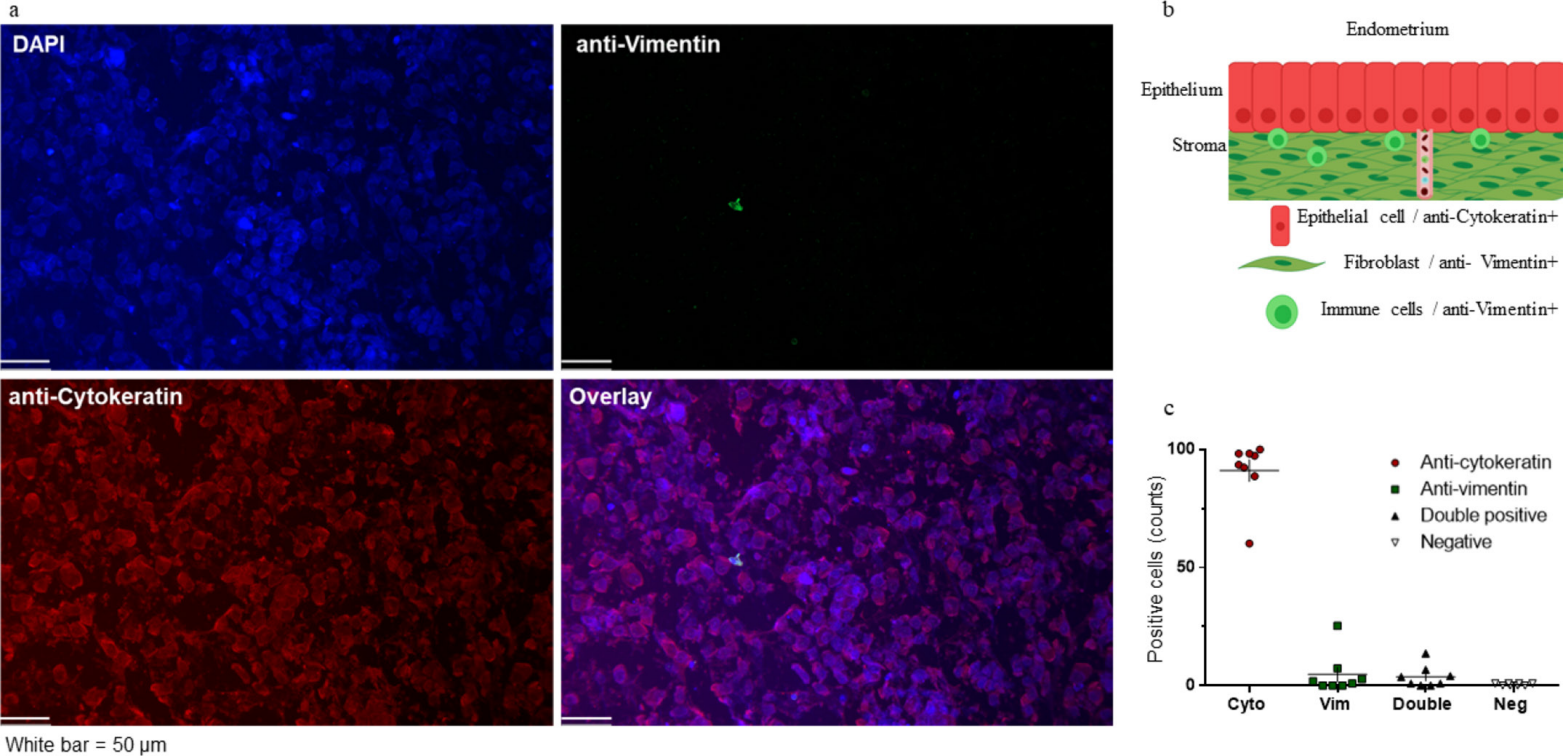
Immunofluorescence labeling of cytobrush-derived luminal endometrial cells. (a) Upright fluorescence microscopy showing 4′,6-diamidino-2-phenylindole (DAPI; nuclear marker), anti-vimentin (mesenchymal cell marker), and anti-cytokeratin (epithelial cell marker) staining of dispersed fresh cells. (b) Graphical representation of the expected staining upon vimentin and cytokeratin analysis, and their respective localizations in the endometrium. (c) Mean ± SEM counts of cells in each animal for each respective marker. Each data point represents one animal (n = 8).

Each brush yielded approximately 10^6^ fresh cells and was used to plate about 3 to 4 wells. It took around 7 to 9 d after collection for the monolayers to reach confluence. Diff-Quik staining showed cell clumps with polygonal conformation, similar to BUECs (Figure 1b). Cell morphology was maintained throughout the 4 passages. There was an effect of passage on the abundance of *IFNAR1* and *VIM* (*P* < 0.05). The abundance of *IFNAR1* was about 6-fold greater in fresh samples than in plated, passage 2, and passage 4 samples. The abundance of *VIM* transcript was greater in passage 2 than in fresh samples. There was no effect of passage on transcription of the epithelial marker *KRT18* (*P* > 0.1).

Treatment of passage 4 BUECs with rbIFN-τ stimulated the expression of *ISG15* and *OAS1* in a dose-dependent fashion (*P* < 0.05; Figure 3). In addition, rbIFN-τ stimulated the expression of *CCL8* and *FABP3*, but only at the highest concentration (*P* < 0.05); rbIFN-τ did not affect transcription of *IRF2*, *IFNAR1*, or *CXCL10* (*P* > 0.1).

**Figure 3:**
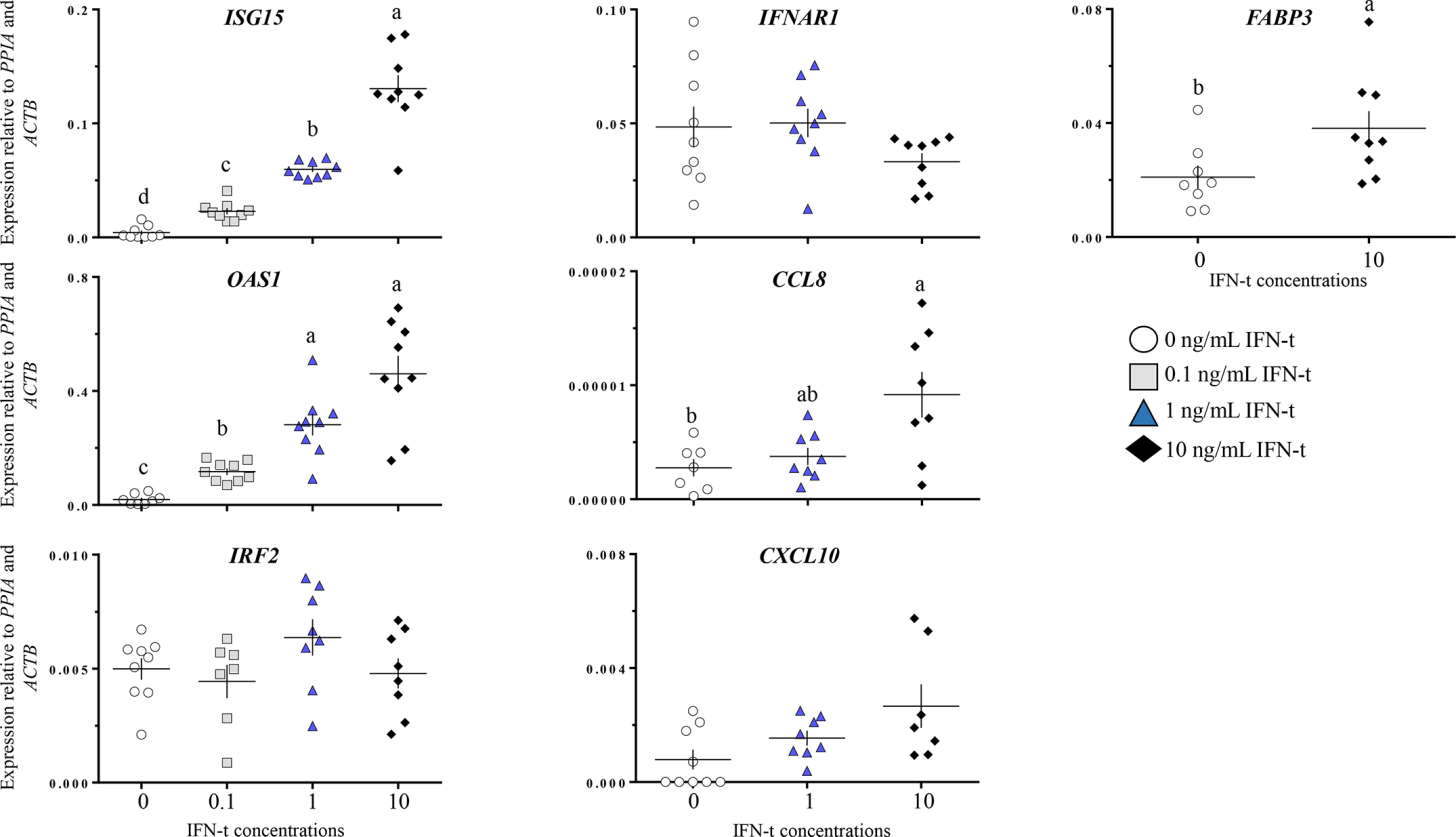
Changes in relative abundance of transcripts in cytobrush-derived endometrial luminal cells (passage 4) treated with increasing concentrations of recombinant bovine IFN-τ (means ± SEM). Each data point represents 1 well. Means with different letters (a–d) are different (*P* < 0.05). Experiment was replicated 3 times; each treatment was administered in triplicate (n = 9/treatment).

The present study demonstrated the viability of using a cytobrush to harvest uterine luminal samples for studies in vivo. Sufficient numbers of luminal epithelial cells were obtained to perform cell culture. Importantly, these cells responded to rbIFN-τ, which is a critical embryo-derived stimulus. Therefore, this is a novel model to study the physiology of endometrial luminal cells in dairy and beef cows in vitro.

The endometrial cells harvested by cytobrush were epithelial in nature, which was confirmed by immunofluorescence and Diff-Quik staining. The predominance of epithelial cells was further indicated by the morphologic evaluation of cells by light microscopy during culture and by the maintenance of *KRT18* relative expression over time. In culture, BUECs underwent a gradual epithelial-mesenchymal transition, as evidenced by an increased expression of VIM. (Sponchiado et al., 2020).

The cytobrush-derived luminal epithelial cells responded to rbIFN-τ. Even with decreased expression of *IFNAR1* through culture progression, signaling was maintained, as evidenced by the stimulation of expression of classical rbIFN-τ targets such as *ISG15* and *OAS1* (Forde et al., 2011; Talukder et al., 2017; Tinning et al., 2020). The integrity of rbIFN-τ signaling was further demonstrated by the stimulation of expression of nonclassical targets such as *FABP3* and *CCL8*, as reported previously (Sakumoto et al., 2017; Schabmeyer et al., 2021). Our inability to detect changes in *IRF2* expression may be related to the timing of our sample collection, as this is an early response gene (Forde et al., 2012).

In conclusion, we demonstrated the feasibility of using cytobrush as a minimally invasive technique to harvest luminal epithelial cells. This approach allows collection of endometrial cells from animals at specific stages of the estrous cycle, which are capable of forming monolayers of mostly epithelial cells. Although the study was performed in *Bos taurus indicus*–influenced beef cows, the findings may be extrapolated to Holsteins and other dairy breeds. Therefore, the current methodology is adequate to study different aspects of endometrial luminal epithelial cell function in cattle, including the IFN-τ response. Additional functional studies, including supplementation with steroid hormones, are warranted to improve the physiological relevance of this system.
